# The unintended consequences of COVID-19 mitigation measures matter: practical guidance for investigating them

**DOI:** 10.1186/s12874-020-01200-x

**Published:** 2021-02-10

**Authors:** Anne-Marie Turcotte-Tremblay, Idriss Ali Gali Gali, Valéry Ridde

**Affiliations:** 1 School of Public Health, Université de Montréal, 7101 Avenue du Parc, Montreal, QC H3N 1X9 Canada; 2 Department and Population, Harvard T.H. Chan School of Public Health, 665 Huntington Avenue, Building 1, Boston, MA 02115 USA; 3 Bureau d’Appui Santé et Environnement, N’Djamena, Chad; 4 IRD (French Institute for Research on Sustainable Development), CEPED, Université de Paris, 45 Rue des Saints-Pères, 75006 Paris, France

**Keywords:** Unintended consequences, Mitigation measures, COVID-19, Pandemic

## Abstract

**Background:**

COVID-19 has led to the adoption of unprecedented mitigation measures which could trigger many unintended consequences. These unintended consequences can be far-reaching and just as important as the intended ones. The World Health Organization identified the assessment of unintended consequences of COVID-19 mitigation measures as a top priority. Thus far, however, their systematic assessment has been neglected due to the inattention of researchers as well as the lack of training and practical tools.

**Main text:**

Over six years our team has gained extensive experience conducting research on the unintended consequences of complex health interventions. Through a reflexive process, we developed insights that can be useful for researchers in this area. Our analysis is based on key literature and lessons learned reflexively in conducting multi-site and multi-method studies on unintended consequences. Here we present practical guidance for researchers wishing to assess the unintended consequences of COVID-19 mitigation measures.

To ensure resource allocation, protocols should include research questions regarding unintended consequences at the outset. Social science theories and frameworks are available to help assess unintended consequences. To determine which changes are unintended, researchers must first understand the intervention theory. To facilitate data collection, researchers can begin by forecasting potential unintended consequences through literature reviews and discussions with stakeholders. Including desirable and neutral unintended consequences in the scope of study can help minimize the negative bias reported in the literature. Exploratory methods can be powerful tools to capture data on the unintended consequences that were unforeseen by researchers. We recommend researchers cast a wide net by inquiring about different aspects of the mitigation measures. Some unintended consequences may only be observable in subsequent years, so longitudinal approaches may be useful. An equity lens is necessary to assess how mitigation measures may unintentionally increase disparities. Finally, stakeholders can help validate the classification of consequences as intended or unintended.

**Conclusion:**

Studying the unintended consequences of COVID-19 mitigation measures is not only possible but also necessary to assess their overall value. The practical guidance presented will help program planners and evaluators gain a more comprehensive understanding of unintended consequences to refine mitigation measures.

**Supplementary Information:**

The online version contains supplementary material available at 10.1186/s12874-020-01200-x.

## Background

In March 2020, the World Health Organization (WHO) declared COVID-19 a pandemic and called for governments to take immediate actions. Its rapid spread led governments and organizations to adopt unprecedented response measures deemed promising (e.g. hygiene, lockdowns, school closures, tracking applications, travel restrictions, financial and psychosocial supports, mass communications). In an effort to saves lives, many healthcare services were postponed while others were revolutionized by removing barriers for medical innovation, introducing technology and changing work patterns [1, 2]. However, the processes and outcomes of implementing innovative solutions are not always as simple and linear as envisioned by program planners. Quickly, the realization that such measures could trigger unintended consequences going beyond their targeted objectives became a topic of discussion among researchers, politicians, journalists, and the population at large [2–8]. Unintended consequences are changes brought by an intervention other than those it aims to achieve [9]. They can be far-reaching and just as important as the intended consequences. They can affect all groups of the population (e.g. service providers, community members, vulnerable people) as well as all sectors of society (e.g. health, education, environment, economy, law). Depending on stakeholders’ perspectives, such unintended consequences can be viewed as desirable, undesirable, or neutral.

### Emerging evidence

There is scientific evidence suggesting that COVID-19 mitigation measures can trigger a wide range of desirable and undesirable unintended consequences within and outside of healthcare systems [10, 11] (see Additional file 1). In the healthcare sector, stakeholders are concerned that a one-track focus on COVID-19 will sow the seeds of other major health crises, such as measles and polio [12]. A modelling study suggested that reductions in coverage of basic life-saving interventions of around 15% for six months would lead to 253,500 additional child deaths and 12,190 additional maternal deaths, across the 118 countries considered, as a result of unavoidable shocks, health system collapse, or choices made in responding to the pandemic [13]. Access to medication has also been affected by COVID-19 mitigation measures. For example, McBirney et al. [14] explained that the “*very discussion of chloroquine and hydroxychloroquine as therapeutic options against COVID-19 has decreased their availability for proven treatments, exacerbated global shortages, fueled an already rampant counterfeit drug market in Africa and worsened trade tensions*.” Although these drugs’ efficacy to treat COVID-19 patients was not proven, some pharmacies in West Africa reported soaring price inflations, stockouts, and the emergence of informal market supply channels [14, 15]. With regard to mental health, reports from multiple countries show increases in domestic violence and alcohol consumption since the COVID-19 outbreak [16]. Stress, the disruption of social and protective networks, loss of income, and decreased access to services can exacerbate the risk of violence and social tensions [16].

Inequitable response measures have also exacerbated ethnic and gender disparities. In France, for example, the rise in deaths was more than twice as high for people born abroad (especially in Africa and Asia) than those born in the country [17]. Researchers in North America are showing that people associated with minority groups are more often classified as “essential workers” and, as such, are unable to work from home, leave their job, or access paid sick leave [18]. They live in denser housing and more polluted areas, which puts them at greater risk during a pandemic [18]. When they do get sick, their access to healthcare is more limited, leading to higher death tolls [18].

Emerging evidence shows that the unintended consequences of COVID-19 mitigation measures go beyond the health sector. The environment has also been affected. On the positive side, contingency policies have been linked to improved air quality, cleaner beaches, and a lessening of environmental noise [19, 20]. Negative side effects have included more waste and less recycling [19]. In the legal field, experts warn that COVID-19 mitigation measures are having negative impacts on civil rights related to privacy (e.g. use of tracking applications), mobility (e.g. travel bans), assembly (e.g. fines for gatherings, including groups of homeless people), and religion (e.g. closure of places of public worship) [21].

### The neglect of unintended consequences in research and evaluation

Historically, researchers and evaluators in public health have neglected the systematic assessment of unintended consequences, despite their breadth and scope [22–25]. To illustrate this tendency, Jabeen [9] searched the database of the DAC Evaluation Resource Centre (DEReC). This is an extensive database of evaluation documents from more than 30 bilateral and multilateral aid providers, although the specific number of documents is not available. Yet, in searching the documents produced in the decade up to July 2012, the author found that only 24 evaluation reports, undertaken or commissioned by 12 different agencies, referred to either unintended or unanticipated consequences. While some of these agencies acknowledged the importance of incorporating strategies to evaluate unintended outcomes in their design, this rhetoric was not translated into evaluation practices. Similarly, a review of program evaluations for the United States Agency for International Development (USAID) showed only 15% of those evaluations considered “unpland/unanticipated results” [26], a decrease from previous years. More recently, de Alteriis [27] conducted an automated textual analysis to determine whether monitoring reports and evaluations from USAID’s Development Experience Clearinghouse (DEC) had considered and/or reported on unintended consequences. Of 1369 reports and evaluations for USAID-funded foreign assistance programs, only 36 had reported on unintended consequences, spread across 20 countries. The author concluded that the results of the review were disappointing.

Numerous explanations have been put forward for the lack of attention to unintended consequences in research and evaluation. One key explanation is that researchers and program planners have difficulty predicting, measuring, and responding to unintended consequences. The usual survey research methods are not always appropriate for investigating innovation consequences [28]. According to de Alteriis [27], additional training and practical tools are needed to answer questions on unintended consequences. Thus far, the training of most practitioners and researchers is aimed mainly at assessing effectiveness, defined as “*the extent to which a given development intervention’s objectives were achieved, or are expected to be achieved …*” [29]. Heider [30] explains that “*the way effectiveness has been defined has kept attention focused on intended results. Most evaluations grapple with getting evidence to determine whether objectives were achieved and to measure an intervention’s contributions. Fewer evaluations are able to collect evidence on effects outside the immediate results chain and identify unintended consequences*.” Consequently, practitioners and researchers have not developed the reflex of considering unintended consequences over time. Other reasons offered to justify the lack of attention to unintended consequences include: the common assumption that innovations or development efforts produce only beneficial results (i.e., pro-innovation bias, paternalistic bias); time and budget constraints; and conflicts of interests of funders and program planners [9, 28, 31–34].

### Why investigate unintended consequences of COVID-19 mitigation measures?

There are numerous reasons for studying the unintended consequences of COVID-19 mitigation measures. First, the likelihood that complex measures would trigger desirable or undesirable unintended consequences going well beyond the objectives of the intervention is high. There is much uncertainty about how mitigation measures with multiple interacting components that target multiple groups and organizational levels will actually unfold in complex systems [28, 35, 36]. According to Woolcock [35], the uncertainty surrounding a complex intervention is related to the numerous pathways and feedback loops involved, the intervention’s exposure to exogenous influences, and the actors’ capacity to exercise discretion (e.g. to act independently of rules or in accordance with self-interest). Researchers argue that engaging with such complexity requires paradigm shifts to carefully consider unintended consequences [36] as well as the big picture of COVID-19 [37].

Another reason for studying unintended consequences is to inform decision-makers. The breadth and scope of unintended consequences may be just as important as the intended consequences. Thus, to be able to judge the overall value of an innovative measure, stakeholders must have a comprehensive understanding of both intended and unintended consequences. With full knowledge of the evidence in hand, program developers and implementers may be able to plan more effective measures by capitalizing on desirable unintended consequences or by altering their strategies to mitigate undesirable ones [9].

There are also economic reasons to study unintended consequences of mitigation measures. Bamberger et al. [31] explain that funding agencies that ignore unintended consequences may continue to support programs that are not achieving their objectives, or are doing so inefficiently. According to Norton [38], unintended consequences can add so much to the costs of some programs that they make them unwise, even if they achieve their stated goals. This has become a central debate during the COVID-19 pandemic, as stakeholders have expressed concerns that undesirable unintended consequences of lockdowns – such as the increase in suicides and post-COVID mental health crises – may outweigh the benefits of preventing the spread of the virus. In this respect, some politicians and researchers have insisted the cure should not be worse than the treatment itself [39, 40].

It is also important to highlight the ethical and legal reasons for studying unintended consequences [34]. Rogers [28] argues that change agents are responsible for the consequences of the innovations they introduce. This recalls the admonition to *do no harm* [41]. According to Mittelmark [25], large-scale interventions that inject a new agenda, money, and people into a setting might disturb it in unplanned ways. Some of the effects may be seriously untoward. In the context of the COVID-19 pandemic, for example, lockdown measures aiming to slow down the transmission of the virus may infringe on basic human rights by causing famines of “biblical proportions” in more than 30 countries [42, 43]. Monitoring the environment for unplanned effects is the external change agent’s minimal ethical obligation to ensure interventions do not cause more harm than good to populations [9, 25]. On a more practical level, it can also help governments and organizations avoid lawsuits filed for infringement of human rights, physical and mental health problems, deaths, financial losses, and bankruptcies resulting from mitigation measures.

### An emerging research agenda for COVID-19

In the context of the COVID-19 pandemic, the importance of assessing the breadth and scope of unintended consequences has received some recognition. For example, WHO has published a Global Research Roadmap that explicitly identifies the assessment of unintended consequences as a top priority [44]. This roadmap highlights the need for research on unintended consequences of: 1) quarantine and isolation; 2) restriction of movement of healthy exposed and infected persons to prevent secondary transmission; and 3) methods used to influence compliance with interventions during outbreak response (e.g. overuse of fear). Unfortunately, however, the roadmap does not provide clues on how best to study the unintended consequences of such mitigation measures.

Some funding agencies such as the Canadian Institutes of Health Research [45] have launched calls for proposals aimed at assessing the *indirect* impacts on individuals and communities within and across jurisdictions globally. In the same vein, the Peter Wall Institute for Advanced Studies offered funding to working groups using unconventional approaches to understand how COVID-19 affects our society in areas such as personal privacy, higher education, and religion [46]. The German Research Foundation (DFG) also launched a call for international multidisciplinary research on the impacts of mitigation measures on a broad range of outcomes [47]. Such funding opportunities may promote research on unintended consequences.

### Lack of guidance on how to assess unintended consequences

Jabeen [9] reviews various evaluation approaches that hold potential to uncover unintended program effects. That review highlights the numerous shortfalls of current evaluation approaches used to assess unintended consequences, including lack of clarity regarding types of unintended effects and insufficient elucidation of methodological guidelines. The author concludes that “*evaluation theory is clearly under-developed regarding examination of unintended effects … [and] previous approaches do not provide sufficient theoretical and empirical guidance for practising evaluators*” [9]. To overcome this gap, the author proposes a three-step process to study unintended consequences: a) outlining program intentions; b) forecasting likely unintended effects; and c) mapping the anticipated and understanding the unanticipated unintended outcomes. While this process is useful, we found that the practical guidance presented is still limited and could be further developed to orient researchers and evaluators in facing the numerous challenges of this field of research.

### Objective of this article

The paucity of theoretical and methodological guidelines for the investigation of unintended consequences has contributed to its neglect in research and evaluation. More practical guidance is needed to support its more systematic integration in research and evaluation. In this article, we argue that it is possible to study the unintended consequences of complex mitigation measures and systems to assess their overall value comprehensively. The objective of the article is to use our past research experiences to share insights and practical guidance for researchers who want to study the unintended consequences of COVID-19 mitigation measures and other complex health interventions and systems.

## Main text

### Background research experiences

Between 2014 and 2020, our team studied the unintended consequences of a complex health intervention in a low-income setting, from which we drew lessons. The results of the study have been published and are available online [48–52]. We began by reading some key literature on the assessment of unintended consequences [28, 53–58]. Based on this literature, we developed a protocol for a study financed by various funding agencies. Our approach was then trialed in a multi-site (*n* = 9) and multi-method study that combined observation in situ, semi-structured interviews, informal discussions, and quantitative routine data. Throughout the study, we engaged with reflexivity by critically analyzing the assessment of unintended consequences on the basis of our experiences to develop new understandings that could ultimately influence our actions [59]. In line with Alexander et al.’s framework [60], we conducted reflexivity *in* action, *on* action, and *underlying* our action in order to improve contemporary research in this field. As such, the first author kept a research journal to record daily reflections on the methods used and their application [61]. This reflexivity enabled us to develop lessons and practical guidance that can be useful for other researchers who want to engage in this type of research. The analysis presented in this article is based on the information collected throughout the study’s development, implementation, and dissemination processes. Our study was conducted in parallel to Jabeen’s [62] work on unintended consequences, so we find it useful to draw on that author’s experience while providing more comprehensive insights to guide research in this area, particularly in the context of the COVID-19 pandemic. To expand beyond our own research experience and present diverse perspectives, we also refer to key literature on the assessment of unintended consequences.

### Considerations to study unintended consequences of COVID-19 mitigation measures

We developed a series of considerations that can be useful when designing and conducting studies on the unintended consequences of mitigation measures. In the subsections below, we discuss each of these considerations in sequence. For illustrative purposes, Table 1 summarizes them using the example of school closures, a mitigation measure widely implemented across the world despite the important ramifications on society and the lack of evidence regarding its efficacy [63, 64]. Since the table has no other objective than to provide an example, empirical research on the unintended consequences of school closures and other mitigation measures (e.g., schools reopening with a mixture of in-class/distance learning) is warranted.

**Table 1:** How to study the unintended consequences of school closures intended to prevent the spread of coronavirus

Considerations to study unintended consequences	Illustrative example
1. Set an explicit objective or research question targeting unintended consequences	How is the nature and implementation of school closures interacting with local actors and the context to trigger unintended consequences?
2. Choose and define your terminology	Unintended consequences are changes for which there is a lack of purposeful action or causation that occur as a result of closing schools.
3. Adopt a theory or conceptual framework	Use a conceptual framework based on Rogers’ *diffusion of innovations theory* to examine how the nature of school closures and their implementation will interact with the social system and the characteristics of its members to produce various types of consequences: 1) undesirable/unanticipated (e.g. negative surprises); 2) undesirable/anticipated (e.g. trade-offs); 3) desirable/unanticipated (e.g. serendipities); 4) desirable/anticipated (e.g. positive spillovers).
4. Determine the study’s perspective	Classify a consequence as anticipated if program planners explicitly referred to it (in documentation or interviews), if it was previously reported in the literature, or if the research team was able to forecast it. Determine whether the consequences are desirable or undesirable, depending on whether the effects of school closures were functional or dysfunctional for the social system.
5. Clarify the intervention theory	Outline program intention by: 1) reviewing program documents and social science theory; 2) interviewing stakeholders; 3) analyzing the discourse of decision-makers in the media; and 4) developing or reviewing the program theory.
6. Forecast potential unintended consequences	Identify a preliminary list of potential unintended consequences of school closures to provide a starting point for data collection (e.g. increased television time, widened social inequalities, increased parental stress).
7. Focus on desirable, undesirable, and even neutral unintended consequences	To avoid a negative bias, collect data on consequences that are desirable (e.g. explore positive new activities, bond with parents), undesirable (e.g. child abuse), and even neutral (e.g. less structured schedules).
8. Include flexible, exploratory methods	Provide families with small wearable cameras during daily activities; ask parents to record diaries using voice memos; conduct participant observation on Facebook groups for parents and in outdoor play areas. Volunteer in a food distribution center for families to conduct observation and build relations with families. Conduct in-depth interviews with stakeholders.
9. Cast a wide net during the data collection	Think broadly and deeply about the wide range of consequences resulting from school closures (e.g. mental, physical, social, financial, developmental, legal) on all members (e.g. children, parents, teachers) of the social system.
10. Follow the evolution of unintended consequences over time	Adopt a longitudinal approach by collecting data related to the different phases of school closures (before, during, after).
11. Adopt an equity lens	Consider differential effects on vulnerable populations, such as children of lower socioeconomic status, from ethnic minorities, or with learning disabilities.
12. Validate the classification of intended versus unintended consequences with stakeholders	Discuss with public health officials, school board representatives, and teachers to validate findings and ensure that the consequences labelled by the research team as unintended were not actually intended.

#### Set an explicit objective or research question targeting unintended consequences

Few studies and evaluations explicitly aim to study unintended consequences of complex interventions from the outset [9, 22–26]. As Patton [53] describes, the study of unintended consequences is often conducted “*if-we-get-to-it-and-have-time-and-resources-after-everything-else-is-done*”. To avoid pious wishes, it is useful to elaborate explicit research objectives and questions regarding unintended consequences in the initial research protocol. This increases the likelihood that sufficient resources will be dedicated to the issue. Having an explicit objective may also encourage the use of appropriate and timely data collection methods and ensure the data are analyzed with this objective in mind. For example, researchers can pose the following research question: How are the nature and implementation of COVID-19 mitigation measures interacting with the local context and members of the social system to produce unintended consequences?

Even if such a research question is not posed at the outset, it is still possible to examine unintended consequences by conducting post hoc analyses. In one study on which the first author collaborated, data revealing unintended consequences emerged from the field during the data collection. Consequently, the research team was compelled to address the issue even though it was not in their initial objectives [65]. Researchers in this situation may have to re-analyze their data to meet this new objective, which ultimately may turn out to be more time-consuming and costly. For such post hoc analyses, it is also possible that not all unintended consequences are reflected in the data, given that the researchers were not actively collecting data on the topic. As a popular axiom says, what we see depends on what we look for [66].

#### Choose and define your terminology

In his seminal article, “The Unanticipated Consequences of Purposive Social Action,” Merton deplored the diversity of terms used to address the issue of unanticipated consequences, which tends to “*obscure the definite continuity in its consideration*” [23]. More than 80 years later, the terminology used to represent this research area continues to vary greatly, indicating a lack of conceptual clarity among researchers [62]. Reviews of the literature on the unintended consequences of health interventions have identified dozens of terms in relation to notions of “unintended” and “consequences” [52, 62]. Based on these reviews, we conducted an exploratory search in PubMed to identify the terms that are used in relation to the unintended consequences of COVID-19 mitigation measures. Table 2 presents the vast array of terms used interchangeably or with fuzzy definitions in the literature on COVID-19. Teasing apart the connotations and research implications of each term can be daunting for researchers or program evaluators. Yet the selection of a specific term can change the analytical lens, creating blind spots that can overlook some pertinent areas. To adequately conceptualize the research object, it is important to select, define, and use the terms representing unintended consequences consistently. This will influence the researchers’ focus during the data collection and analyses, as well as make the findings clearer.

**Table:** Box 1 Definition of unintended consequences

We propose using the term “unintended consequences”, defined as changes for which there is a lack of purposeful action or causation that occur to a social system as a result of the adoption and implementation of an intervention. [28, 86]

**Table 2:** Terms used in relation to unintended consequences of COVID-19 mitigation measures

Concept 1 - “Unintended”	Concept 2 - “Consequence”
Unintended [67]	Consequence [67]
Unanticipated [68]	Effect [69, 70]
Collateral [69, 71]	Risks [68]
Side effects [70]	Harm [41, 72, 73]
Unexpected [74]	Disruptions [74]
Unforeseen [75]	Impact [76]
Spillover [77]	
Ripple [78]	
Typhoon eye [79]	
Undesirable [80]	
Unwanted [81]	
Detrimental [76]	
Indirect [19]	
Wider [82, 83]	
Downstream [80]	

Based on our experience [49], we argue that using the term “unintended consequences” has the following advantages. First, the neutrality of the word “unintended” enables researchers to include desirable (positive), undesirable (negative), and neutral changes in their analysis. While the term “collateral” appears to have gained considerable traction in the literature on COVID-19, it has a negative connotation due to its common usage in reference to injury or damage to civilians during war. This term may dispose researchers to focus primarily on negative consequences. Second, the word “unintended” enables researchers to include changes that are both anticipated and unanticipated by program planners. Effects that are not intended might very well be anticipated by program planners or researchers [11, 84]. Third, the word “consequences” enables researchers to focus not only on the processes, but also on the effects or impacts of innovations. Changes can emerge at any point in time during the cycle of the mitigation measures.

#### Adopt a theory or conceptual framework

Theories and analytic frameworks are known to increase quality and rigour in science [85]. Thus far, we have noticed that most articles related to the unintended consequences of COVID-19 mitigation measures do not make explicit references to either theories or frameworks. According to Jabeen [62], theories can help: 1) predict unintended outcomes and the likely mechanisms generating them; 2) explain findings about unintended effects and the mechanisms producing them; and 3) attribute identified unintended outcomes to the program.

**Fig. 1:**
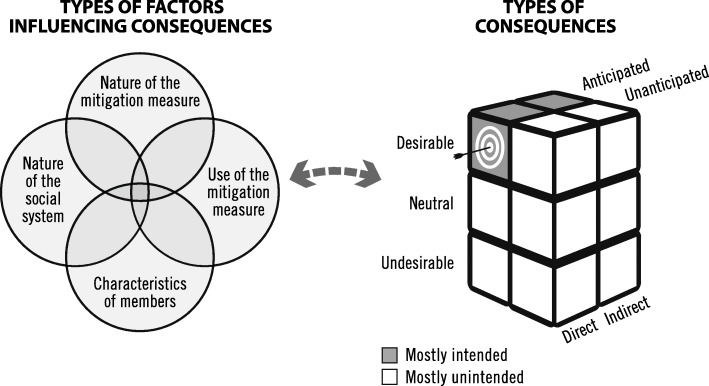
Framework for studying unintended consequences. Adapted from Rogers [28], Bloomrosen et al. [86] and Turcotte-Tremblay et al. [48–50]

Selecting a theory to study unintended consequences can be difficult, but there are several possibilities. Our team used literature on the diffusion of innovations theory to help develop the framework illustrated in Fig. 1 [28, 48–50, 86].

According to this framework, four categories of independent variables can interact to influence the consequences of new mitigation measures. First, it is important to consider the *nature of the mitigation measures*. This refers to their attributes, such as their relative advantage, compatibility with local needs, and complexity [28]. Second, researchers should consider the *characteristics of members of the social system* to understand the unintended consequences that emerge. Such characteristics include their socioeconomic status, type of job, health status, and access to slack resources during the pandemic, as well as their perceptions and attitudes towards mitigation measures. Third, it is important to examine the *nature of the social system* within which the mitigation measure is introduced in order to understand its consequences. This includes local norms as well as the inner and outer environment. Fourth, the *implementation* of mitigation measures also influences the consequences that emerge. For example, the use of police brutality to enforce lockdowns and curfews in LMICs may have prevented some people from seeking care for essential healthcare services and created mistrust among the population [87].

The consequences arising from mitigation measures can be classified into three dimensions [28]. First, they can be desirable, neutral or undesirable. This depends on whether the effects of a mitigation measure tend to be functional for the social system (i.e., positive, producing additional benefits, helping the system work properly), or dysfunctional (i.e., negative, causing harm, not helping the system work properly). Second, consequences can be direct or indirect, depending on whether the changes to a social system occur as an immediate response to an innovation or as a second-order result of the direct consequences. Third, consequences can be anticipated or unanticipated, depending on whether the changes are recognized by the members of a social system. We consider that the following types of consequences tend to be unintended by program planners: undesirable/anticipated, undesirable/unanticipated, and desirable/unanticipated. Our rationale for classifying these consequences as unintended is that program planners are not likely to purposefully target changes they consider undesirable or that they have not anticipated. Like Bloomrosen et al. [86], we expected that consequences that are desirable/anticipated (e.g. limiting the spread of the virus) would tend to be intended by program planners. As Jabeen [62] argued, program planners trying to promote a mitigation measure are likely to have listed and exhausted all the desirable outcomes that they foresee in the program’s objectives. However, it is important to remain open to the possibility that some desirable/anticipated consequences could be unintended if they were, for example, positive spillover effects that were foreseen but not initially targeted by program planners, such as improvements in air quality following COVID-19 lockdowns. For illustrative purposes, we refer again to the example of school closures to show how potential unintended consequences can be classified (see Table 3). Empirical research will be needed to test these consequences.

**Table 3:**
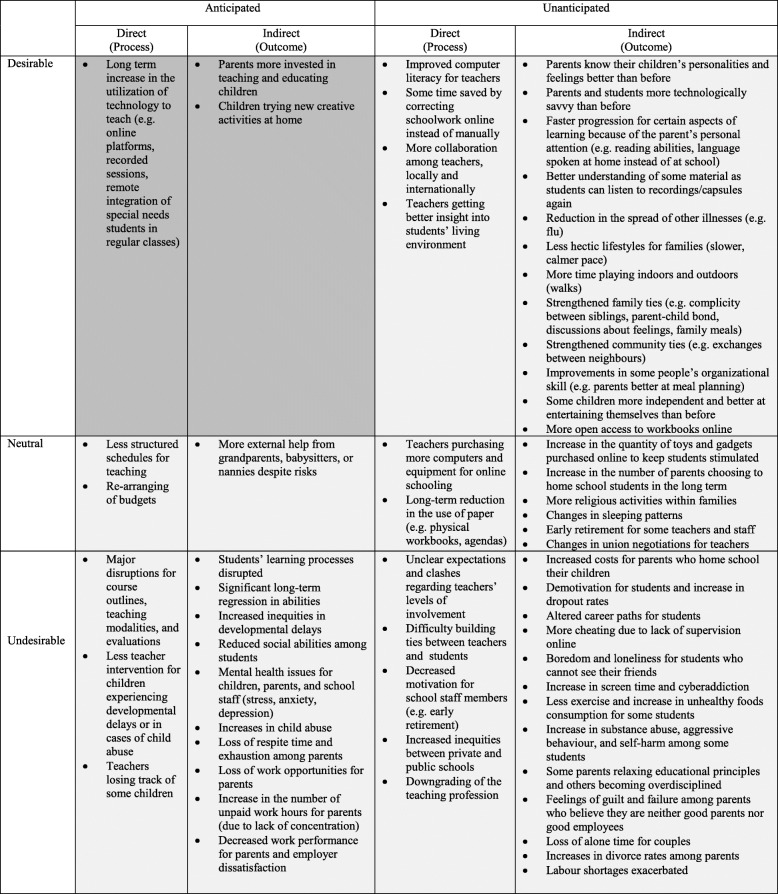
Example of classification of unintended consequences potentially resulting from school closures for COVID-19

Note that intended consequences are not included in this example

According to our framework, the dark and light grey sections indicate sections that may be more likely to include “intended” and “unintended” consequences, respectively

The *complexity approach* also proposes concepts that can be useful to understand the mechanisms by which mitigation measures produce unintended consequences, including feedback loops, the relation between the parts and the whole (systemic principle), the level of fit between an intervention and its context, and the dialogical principle, whereby notions that appear to be contradictory can be part of a unique whole [32, 58].

Moreover, some authors have proposed typologies to classify unintended consequences. In public health, Bloomrosen et al. [86] identified six types of unintended consequences: cognitive, care process, organizational, social/legal, fiscal, technology. These unintended consequences can affect different types of stakeholders, such as patients, providers, organizations, vendors, payers, and governments. More recently, from a literature review on unintended harm in public health interventions, Allen-Scott et al. [10] identified five types of unintended harm: physical; psychosocial; economic; cultural; and environmental.

Taken together, these different typologies can be useful to encourage researchers to consider the broad specifications of unintended consequences that can emerge during data collection and different ways of classifying unintended consequences during the analyses. Adopting an inclusive typology *ex ante* compels researchers and evaluators to consider the possibility that interventions can produce consequences that are not intended, instead of focusing only on intended ones. Alternatively, it is also possible to study unintended consequences by using grounded theory or a more inductive approach that does not rely on existing theories or frameworks during the data collection.

#### Determine the study’s perspective

Researchers must operationalize concepts related to unintended consequences. In doing so, they need to choose a point of view. Who defines what is intended or unintended by an intervention? Based on our experience, we found that stakeholders at the different levels of public service (e.g. decision-makers versus street level workers) may have different understandings of the intended or unintended consequences, thereby adding complexity to their classification.

Similarly, when using the notion of anticipation, researchers should also specify “for whom”. The degree of anticipation of consequences can vary depending on stakeholders’ position or even imagination. For example, researchers familiar with the scientific literature on pandemics are likely to have anticipated certain unintended consequences, such as the plunge in consultations for emergencies unrelated to COVID-19 (e.g. heart attacks). Yet, this was described as “unanticipated” by certain outlets. Some authors decided to deal with this issue differently by classifying unanticipated consequences as “foreseeable” versus “unforeseeable”, depending on whether the changes induced by the intervention *could* have been predicted beforehand, had adequate efforts been made [54, 62].

Moreover, in classifying the level of desirability of unintended consequences, researchers may have to ask the question, “desirable or undesirable for whom?” The level of desirability can vary when the perceptions of decision-makers are compared with those of street-level workers, who have different goals and needs. Operationalizing these concepts imposes difficult choices on research teams, but examples of how to do it are available in the literature [48–50, 65].

#### Clarify the intervention theory

Based on our experience, we found that to correctly identify an intervention’s unintended consequences, researchers and evaluators must first understand the intended processes and outcomes. This can be done by clarifying the intervention theory, which describes how an intervention unfolds and brings about change [85]. This knowledge will help to orient the researchers’ focus during the data collection and analysis. This is akin to Jabeen’s [62] first step in studying unintended consequences, which is to outline the program’s intention by: 1) reviewing program documents and social science theory; 2) interviewing stakeholders; and 3) developing or reviewing the program theory and outlining the intended outcomes.

However, in the context of the COVID-19 pandemic, it is not always possible to review program documents because the “aircraft is built in flight”. In a vortex of uncertainty, decisions and actions are taken quickly, under pressure, with little in the way of preparation or blueprints [21]. Moreover, the justification behind mitigation measures sometimes changes along the way. For example, the government of Quebec (Canada) initially presented deconfinement measures as a way of gaining herd immunity, but then reversed itself on the issue. Thus, researchers may have to outline and consider the adaptations of the program’s intervention theory based on the literature available and decision-makers’ discourse as presented in the media.

It should be noted that researchers using goal-free evaluation remain deliberately unaware of program intentions to avoid a narrow view [9]. Goal-free evaluation leads researchers to investigate actual outcomes that can be either intended or unintended. This approach may be appropriate for researchers who want to simultaneously analyze both intended and unintended outcomes. However, researchers who want to focus only on unintended consequences for various reasons (e.g. knowledge gap, limited resources) should clarify the intervention theory to orient the data collection and analysis as well as to enable consequences to be classified as unintended.

#### Forecast potential unintended consequences

Like Jabeen [62], we find that reviewing the literature is useful to identify a preliminary list of potential unintended consequences that could provide a starting point for data collection. Additional file 1. presents examples of unintended consequences of mitigation measures for COVID-19 found in the literature. To compile this list, we conducted a rapid review of articles available on PubMed with the terms “unintended” and “COVID-19” in their titles or abstracts. We also included a few newspaper articles to illustrate the pertinence of information provided in a timely manner. At the time of writing this paper, the overall strength of the evidence was rather weak. Many findings on unintended consequences were based on newspaper articles or grey literature, and little information was provided on the methods used to collect information.

As literature on the unintended consequences of response measures to COVID-19 is only beginning to emerge, researchers may find it useful to expand their search to examine literature on past epidemics, such as the 2003 outbreak of severe acute respiratory syndrome (SARS) or the 2014 Ebola outbreak, where entire villages in many West African countries were quarantined [88]. For example, one study found that the ban on bushmeat in West Africa during the 2013–2016 Ebola outbreak served to proliferate informal networks of wild animal trade and sale, rendering the development of acceptable, evidence-based surveillance and mitigation strategies for zoonotic spillovers almost impossible [89]. Moreover, Faherty and Doubeni [90] presented preliminary data suggesting that screening procedures for Ebola in the United States could unintentionally add stress from stigma among the West African diaspora. This raises the possibility that COVID-19 and its mitigation measures may have increased stigma and discrimination against specific ethnic communities or groups of the populations that were strongly affected by the disease (e.g. Chinese, Black, or Latino communities, urban dwellers, healthcare workers).

The results of such literature reviews, however, should not limit the researchers’ focus during data collection. Researchers should try to capture unintended consequences that are context specific or unexplored in the literature.

Jabeen [62] also suggests researchers can predict unintended outcomes by interviewing experts and involving stakeholders (e.g. decision-makers, program planners) in forecasting outcomes. While this is true, we did find that, in some cases, local stakeholders have little to say about the potential unintended consequences that could emerge. This may depend on the sensitive nature of the topic, the level of reflexivity among actors, and the stakeholders’ conflicts of interests.

#### Focus on desirable, undesirable, and even neutral unintended consequences

Studies have found that unintended consequences tend to be mostly undesirable or negative [11, 32, 62]. To counterbalance this tendency towards the negative, researchers should collect data on desirable, undesirable, and even neutral unintended consequences. For example, response measures to the COVID-19 pandemic appear to have triggered some desirable unintended consequences, such as improved air quality, cleaner beaches, and less environmental noise, although the net impact on the environment will likely remain negative [19]. In the past, positive unintended consequences of the SARS outbreaks included increased social cohesion among certain groups (e.g. displays of respect for infected professionals, donations for orphans, fund-raising concerts, neighbourhood-initiated cleaning campaigns) [91]. SARS outbreaks also promoted support for family members as well as healthy behaviours such as resting and exercising [92, 93]. Neutral unintended consequences of pandemics can include a strengthened faith in God [92] or a looser sense of time [94]. Explicitly widening the focus of the study to encompass desirable, undesirable, and neutral unintended consequences may help researchers be more readily accepted within a research setting (e.g. healthcare organization, public health agency, school). Local stakeholders may feel less threatened when researchers explain that they will not focus only on negative findings. Moreover, presenting both desirable and undesirable unintended consequences can provide a more complete, balanced picture. When reporting the results, researchers should also report on the expected unintended consequences that did not arise. This may help researchers present more balanced findings to stakeholders.

#### Include flexible, exploratory methods

Researchers cannot foresee all unintended consequences when developing their protocol. Thus, research teams should remain flexible and open during the data collection to capture data on all unintended consequences that emerge. Exploratory qualitative methods, such as those commonly used in anthropology, can be powerful tools to study unintended consequences [28]. In our experience, interview guides and observation grids had to be adapted and refined as unintended consequences became perceptible to the research team over time [48]. We also found that conducting observation in situ over a long period of time can be crucial to study hidden behaviours (e.g. alcohol consumption) or illicit practices (e.g. circumvention measures) in a more natural context [48]. As Jabeen [62] explains, the narratives people tell and the reality of an event do not always align.

In midst of a pandemic, however, traditional data collection methods may have to be adapted. Strict confinement measures may require that researchers develop new creative ways of collecting data on different groups. Online interviews or focus groups can be conducted using platforms such as Skype or Zoom [95, 96]. Digital interviews can be conducted live using WhatsApp [97] or asynchronously using emails [98]. Participants can also report on their personal experience using smartphone cameras, diaries, voice memos, or online platforms or apps [98, 99]. Small wearable cameras can be used during daily activities to conduct “walk-alongs”, “eat-alongs” or “work-alongs” [98]. Videos can then be viewed with participants while asking questions about their experiences.

With the increased use of the Internet and social media around the world, innovative methods such as digital ethnography or nethnography offer rich opportunities to study the unintended consequences of COVID-19 response measures [100, 101]. Miller [102] argues that, during physical isolation, researchers should actually concentrate more on observations than interviews to compensate for the fact that they are not physically present on site. Offering help during the pandemic (e.g. to develop online resources) can be a point of entry to get to know people, build trust, and engage with others online [102]. Salmons [103] explains how different types of observation with varying levels of engagement can be conducted online (e.g. unobtrusive observation, participant observation). Regardless of the method used, researchers should observe long enough to get a sense of repetitivity, typicality, and normativity [102].

#### Cast a wide net during data collection

Researchers must think broadly and deeply about the wide range of consequences resulting from COVID-19 mitigation measures [40]. Questioning participants about the unintended consequences of complex interventions is not always a straightforward task. In our experience, general questions, such as “*did the intervention lead to any unintended consequences?*” or “*did the intervention cause changes that surprised you?*” did not always yield interesting results during semi-structured interviews. Often, respondents simply answered, “no, it did not.” Participants do not always fully comprehend an intervention’s logic model or intended outcomes, especially if the design is complex. Thus, to capture pertinent data, we recommend that researchers cast a wide net by asking a lot of questions about different aspects of the intervention and the various types of changes they triggered. It is important to be creative and think outside the box to detect potential spillover effects that were not expected, even by the research team. Researchers should test different leads and triangulate the data through various sources. This data collection process is analogous to that of a fisherman casting a net widely into the sea to catch fish of every kind. During the data analysis, researchers can conduct a triage and distinguish more carefully between intended and unintended consequences. The main challenge with this approach is that it results in a colossal amount of data on both intended and unintended consequences.

Alternatively, studies can be conducted on specific unintended outcomes of COVID-19 measures. For example, quantitative studies have been conducted on the impact of confinement on lifestyle behaviours in children [71]. This approach might, however, restrict the focus to unintended consequences that can be foreseen by researchers.

#### Follow the evolution of unintended consequences over time

The consequences resulting from COVID-19 measures are likely to evolve over time. Some unintended consequences may be immediate, such as increases in intimate partner violence or child abuse [3]. Others, such as increases in criminality, may only be observable months or years later because of the long-term economic consequences of the pandemic [3]. Moreover, the mitigation measures themselves are likely to evolve over time as the multiple waves of the pandemic unfold. Some may become permanent (e.g. personal hygiene, physical distancing in public spaces), while others have already been modified (e.g. distancing practices among children). Longitudinal approaches may thus be useful to understand the emergence of unintended consequences over time. For example, interrupted time series are powerful designs which have been recommended to evaluate unintended consequences [104].

#### Applying an equity lens

Individuals and communities are affected differently by quarantine, limitations in movement, and other restrictive measures. There is a growing consensus among experts that applying an equity lens is useful to understand the unintended consequences of mitigation measures, particularly on people who are marginalized and face intersecting forms of discrimination, such as elderly people living with disabilities in long-term care housing [21, 40, 105, 106].

Evidence is increasingly showing that mitigation measures are exacerbating various types of social inequalities, particularly related to age, socio-economic status, gender and ethnicity. For example, Clinton and Sridhar argue that we need to turn our attention to children who are at risk of experiencing disproportionate amounts of unintended consequences due to reduced access to life-saving healthcare services [107]. In addition, Quesnel Vallée’s analyses suggest that people living in low-income, high-density areas with employments that offer little protective gear or possibilities to work from home have been disproportionately affected by COVID-19 [105]. In various parts of the globe, ethnic minorities with these underlying living conditions are infected, hospitalized, and killed at a disproportionate rate [105, 108]. Systemic racism has predisposed ethnic minorities to high-risk jobs and lower quality care, which has repercussions on the outcomes of the pandemic [108]. In France, a study found that confinement measures contributed to deepening social and health inequalities for Afro-Caribbean immigrants, as well as forms of resilience [109]. Participants reported widespread fears, exacerbated by police controls, situations of deprivation and weakening of informal support networks. An equity lens should be applied to take into consideration such inequitable implementation problems to assess unintended consequences and tailor interventions appropriately.

In this vein, applying a gender lens to the COVID-19 response can also be useful [106]. Women represent 70% of the health and social sector workforce, which exposes them to greater risks of contracting the disease and of facing discrimination [106]. In times of crisis, women and girls are also at higher risk of intimate partner violence and other forms of domestic violence due to increased tensions in the household [106]. A “pink-collar recession” may also be underway due to the disproportionate economic impacts of lockdowns on women [110]. For many women in healthcare and education, the crisis has made their paid workload greater or more complex [110]. For others, the crisis has decimated their work opportunities and substantially increased their unpaid care work [110].

Concretely, there is some practical guidance that researchers can follow to apply an equity lens to assess the unintended consequences of COVID-19 mitigation measures [111]. First, researchers must ensure marginalized people are seen, heard, and sufficiently considered throughout the research process. Special attention must be paid to vulnerable subgroups when elaborating research questions, data collection methods, and analyses plans. As Yaya et al. [108] explain, the acquisition of disaggregated data is vital in identifying gaps in the social determinants of health disparities and tailoring global policy responses to COVID-19. Second, including members of vulnerable groups on the research team may be useful to understand how COVID-19 impacts them disproportionately. Ridgway [112] has called for more patient-partnered research during the COVID-19 pandemic in order to take “lived experiences” into consideration. While it might take more time to involve patient partners, Ridgway argues that it leads to better research and that there is a cost to pay when researchers neglect contacts with patients. Third, intersectionality-informed analyses can be useful to highlight the complex relationships and interactions between COVID-19, social identities, social inequities, power dynamics, and social context [113, 114].

#### Validate the classification of intended versus unintended consequences with local stakeholders

Jabeen [62] argues that any program outcomes not identified when the program intentions were first outlined should be considered unintended. However, we recommend more flexibility in this regard. Even if researchers diligently try to understand an intervention’s logic model, some of the consequences they identify as unintended may have been considered by program planners to be intended. Thus, to verify their classification, it can be useful to present the findings on unintended consequences to stakeholders while protecting confidentiality [115]. In our experience, some consequences that emerged were clearly identified as intended when we discussed the results with stakeholders, even though they had not been initially identified as such during our preliminary work to outline the intervention’s complex logic model. Maintaining that these consequences were unintended despite this feedback could have discredited the whole study. Second, some interventions are dynamic and have evolving logic models. For the COVID-19 pandemic, governments’ response measures and their justifications evolve daily. Program intentions as initially outlined may no longer be an adequate point of reference to determine what was or was not intended. Thus, researchers should leave open the possibility of adapting to this evolution as the intervention unfolds, as they see fit.

### Discussion on the implications of this approach

This paper has addressed the paucity of theoretical and methodological guidelines for investigating the unintended consequences of innovative measures, such as those adopted during the COVID-19 pandemic.

The approach presented in this paper is likely to be relevant for the coming years. First, experts are expecting additional waves of coronaviruses, as well as other high impact pandemics, in the years to come. Thus, mitigation measures are likely to continue to be imposed to various degrees, raising questions about long-term unintended consequences of repeated disturbances for children, business owners, etc. Second, governments and private companies are searching for innovative strategies to respond to coronaviruses. The development and use of new technology, such as monitoring through smartphone applications and using drones to track the movement of people, are likely to trigger new ethical issues and coping strategies that did not exist in the past, sometimes even blurring the lines between what we previously perceived as reality and fiction [116]. One might even wonder whether the assessment of unintended consequences might eventually become the “new normal” in program evaluation and research.

The systematic assessment of unintended consequences should be a critical component of the response to COVID-19, as it can inform the effective deployment of these strategies. Examples of how the assessment and monitoring of unintended consequences can help refine and develop new mitigation measures have already emerged at the international and national levels. Social stigma against people associated with coronavirus (e.g. health workers, recovered patients, families of infected persons) led UNICEF, WHO and the International Federation of Red Cross and Red Crescent Societies (IFRCRCS) to launch a guide intended to support governments, media, and local organizations to counter stigmatizing attitudes [117]. Similarly, concerns about increasing social inequities led the United Nations to actively promote the adoption of a human rights approach to responding to the pandemic [118]. UN Women published recommendations to prevent and respond to violence against women and girls, at the onset, during, and after the public health crisis, with examples of actions already taken [119]. In Iran, a social media platform was created to help students cope with the anxiety and stress brought on by the COVID-19 pandemic [120]. In Canada, a federal compensation program for people who had lost employment due to the pandemic outweighed the salaries of some essential workers and actually became a disincentive to work, spurring the government to increase the wages of essential service workers to retain them in the workforce [5]. Consideration of the unintended consequences of confinement measures led public health authorities in Quebec to launch a physical activity program to keep seniors active and shore up morale [121]. Alcohol outlets were categorized as essential services and were permitted to remain open to avoid increasing the number of cases of severe alcohol withdrawal syndrome, as was observed in India [122].

The lack of attention to unintended consequences is a general shortcoming in the training and practices of evaluators and researchers. Thus, we believe the insights presented in this paper can also be used to understand the unintended consequences of other complex interventions not related to the COVID-19 pandemic. In the pursuit of sustainable development goals, for example, global health actors are promoting and testing a wide array of interventions that are deemed promising but that will inevitably cause unintended consequences. According to the law of unintended consequences, the actions of people, and especially of governments, always have effects that are unanticipated or unintended [38]. This law is deemed to be “at work always and everywhere” [38].

### Limitations

The practical guidance provided in this paper should be interpreted with caution as it is based on a limited amount of past experiences and literature. The considerations presented should be validated and refined through future research and input from other researchers in this area. A “one size fits all” approach to studying the unintended consequences of COVID-19 mitigation measures is not desirable given the wide breadth and scope of unintended consequences that impact all aspects of life. Our aim is not to standardize research on unintended consequences, but rather to contribute to strengthening practices and reflexivity in this area. We hope this paper will stimulate dialogue and exchanges that can promote the development of methods to study unintended consequences.

## Conclusion

There is currently a lack of clarity and consensus about the best practices to study unintended consequences of health interventions, which has contributed to the paucity of research on this topic. However, in this paper we have shown that theories and methods to understand the unintended consequences of COVID-19 measures do exist. We have presented some practical guidance to support the development of this approach. To judge the overall value of mitigation measures, program planners, researchers, and evaluators increasingly need to understand unintended consequences. It is our hope that the practical guidance presented here may inspire researchers wishing to explore the unintended consequences of COVID-19 mitigation measures, which have already had, and continue to have, impacts on every aspect of life across the globe.

## Twitter

Anne-Marie Turcotte-Tremblay @AnneMarieTrem and Valéry Ridde @ValeryRidde.

## Supplementary Information

**Additional file 1.**

## Data Availability

Data sharing is not applicable to this article as no datasets were generated or analyzed during the current study.

## Abbreviations

COVID-19: Coronavirus disease 2019
DEC: Development Experience Clearinghouse
LMICs: Low- and middle-income countries
UNDP: United Nations Development Programme
USAID: United States Agency for International Development
WHO: World Health Organization

## References

[1] Wang X, Bhatt DL (2020). COVID-19: an unintended force for medical revolution?. J Invasive Cardiol.

[2] English M, Moshabela M, Nzinga J, Barasa E, Tsofa B, Marchal B, et al. Systems and implementation science should be part of the COVID-19 response in low resource settings. BMC Med [Internet]. 2020 Jul 15 [cited 2020 Dec 15];18. Available from: https://www.ncbi.nlm.nih.gov/pmc/articles/PMC7360474/. 10.1186/s12916-020-01696-6 PMC7360474 32664950

[3] INSPQ (2020). COVID-19 – Exploration des indicateurs de suivi de la violence, de la sécurité, du sentiment de sécurité, de la criminalité et des tensions sociales [Internet].

[4] Jung A. Unintended consequence of COVID-19: Fewer parents taking kids to get immunized. CTV news [Internet]. 2020; Available from: https://bc.ctvnews.ca/unintended-consequence-of-covid-19-fewer-parents-taking-kids-to-get-immunized-1.4922332.

[5] Labbé J. COVID-19 : Québec veut augmenter le salaire de certains travailleurs essentiels. Radio-Canada. 2020; Available from: https://ici.radio-canada.ca/nouvelle/1690832/programme-incitatif-retention-pirte-coronavirus.

[6] Sharma S, Lawrence C, Giovinazzo F. Transplant programmes during COVID-19: unintended consequences for health inequality. Am J Transplant. 2020. 10.1111/ajt.15931 PMC9800440 32314551

[7] Weissgerber T, Bediako Y, de Winde CM, Ebrahimi H, Fernández-Chiappe F, Ilangovan V (2020). Mitigating the impact of conference and travel cancellations on researchers’ futures. Elife..

[8] Paul E, Ridde V. Évaluer les effets des différentes mesures de lutte contre le Covid-19, mission impossible ? [Internet]. The Conversation. [cited 2020 Jun 7]. Available from: http://theconversation.com/evaluer-les-effets-des-differentes-mesures-de-lutte-contre-le-covid-19-mission-impossible-135060.

[9] Jabeen S (2016). Do we really care about unintended outcomes? An analysis of evaluation theory and practice | Elsevier enhanced reader. Evaluation and Program Planning.

[10] Allen-Scott LK, Hatfield JM, McIntyre L (2014). A scoping review of unintended harm associated with public health interventions: towards a typology and an understanding of underlying factors. Int J Public Health..

[11] Koch D-J, Schulpen L. Introduction to the special issue ‘unintended effects of international cooperation.’ Evaluation and Program Planning. 2018 Jun 1;68:202–209. 10.1016/j.evalprogplan.2017.10.006 29029803

[12] MSF. COVID-19 Must Not Jeopardise the fight against major killer diseases. In DRC, the measles outbreak is far from over [Internet]. 2020. Available from: https://msf.exposure.co/measles-in-drc.

[13] Roberton T, Carter ED, Chou VB, Stegmuller A, Jackson BD, Tam Y, et al. Early Estimates of the Indirect Effects of the Coronavirus Pandemic on Maternal and Child Mortality in Low- and Middle-Income Countries [Internet]. Rochester, NY: Social Science Research Network; 2020 Apr [cited 2020 May 19]. Report No.: ID 3576549. Available from: https://papers.ssrn.com/abstract=3576549. 10.1016/S2214-109X(20)30229-1 PMC7217645 32405459

[14] McBirney S, Baxi S, Kumar KB, Richmond T. The unintended consequences of a proposed cure for COVID-19. TheHill [Internet]. 2020 Apr 29 [cited 2020 May 1]; Available from: https://thehill.com/opinion/healthcare/495248-the-unintended-consequences-of-a-proposed-cure-for-covid-19.

[15] CORAF. Chloroquine self-medication for the coronavirus: Warning [Internet]. 2020. Available from: https://www.sonar-global.eu/wp-content/uploads/2020/03/CRCF-Note-on-Chloroquine-for-COVID-210320.pdf.

[16] WHO. COVID-19 and violence against women. What the health sector/system can do [Internet]. 2020. Available from: https://www.who.int/reproductivehealth/publications/emergencies/COVID-19-VAW-full-text.pdf.

[17] Papon S, Robert-Bobée I. Une hausse des décès deux fois plus forte pour les personnes nées à l’étranger que pour celles nées en France en mars-avril 2020 - Insee Focus - 198 [Internet]. Insee; 2020 [cited 2020 Jul 7]. Report No.: 198. Available from: https://www.insee.fr/fr/statistiques/4627049.

[18] Resnick B. Police brutality is a public health crisis. Vox [Internet]. 2020 [cited 2020 Jun 7]; Available from: https://www.vox.com/science-and-health/2020/6/1/21276828/pandemic-protests-police-public-health-black-lives-matter.

[19] Zambrano-Monserrate MA, Ruano MA, Sanchez-Alcalde L (2020). Indirect effects of COVID-19 on the environment. Sci Total Environ.

[20] Dang H-AH, Trinh T-A (2020). Does the COVID-19 lockdown improve global air quality?. New cross-national evidence on its unintended consequences Journal of Environmental Economics and Management.

[21] Flood C (2020). Collateral damage: the unintended consequences of COVID-19.

[22] Bonell C, Jamal F, Melendez-Torres GJ, Cummins S (2015). ‘Dark logic’: theorising the harmful consequences of public health interventions. J Epidemiol Community Health.

[23] Merton RK (1936). The unanticipated consequences of purposive social action. Am Sociol Rev.

[24] Morell JA (2005). Why are there unintended consequences of program action and what are the implications for doing evaluation?. Am J Eval.

[25] Mittelmark MB (2014). Unintended effects in settings-based health promotion. Scand J Public Health.

[26] Hageboeck M, Frumkin M, Monschein S. Meta-evaluation of quality and coverage of USAID evaluations 2009–2012 [Internet]. 2013. Available from: http://www.ghpro.dexisonline.com/sites/default/files/Meta-Evaluation%20of%20Quality%20and%20Coverage%20of%20USAID%20Evaluations%202009-2012.pdf.

[27] de Alteriis M. What can we learn about unintended consequences from a textual analysis of monitoring reports and evaluations for U.S. foreign assistance programs? Evaluation and Program Planning [Internet]. 2020 [cited 2020 May 7];79. Available from: https://reader.elsevier.com/reader/sd/pii/S0149718919303192?token=72D834BDFA64245C28C996001E951FF029EC299C74888875A04357B675F34ED0454E213F9B7EF053FEE03D87811CEB7D. 10.1016/j.evalprogplan.2020.101779 31981927

[28] Rogers EM (2003). Diffusion of innovations.

[29] OECD. Glossary of Key Terms in Evaluation and Results Based Management [Internet]. OECD PUBLICATIONS. Paris:France; 2002. Available from: http://www.oecd.org/dac/evaluation/2754804.pdf.

[30] Heider C (2017). Rethinking evaluation - what is wrong with development effectiveness? [internet].

[31] Bamberger M, Tarsilla M, Hesse-Biber S (2016). Why so many “rigorous” evaluations fail to identify unintended consequences of development programs: how mixed methods can contribute. Eval Program Plann.

[32] Morell JA (2018). Systematic iteration between model and methodology: a proposed approach to evaluating unintended consequences. Eval Program Plann..

[33] Oliver K, Lorenc T, Tinkler J (2019). Evaluating unintended consequences: new insights into solving practical, ethical and political challenges of evaluation. Evaluation..

[34] McQueen DV (2014). Evidence and harm: time for reflection. Int J Public Health.

[35] Woolcock M (2013). Using case studies to explore the external validity of ‘complex’ development interventions. Evaluation..

[36] Greenhalgh T, Papoutsi C. Studying complexity in health services research: desperately seeking an overdue paradigm shift. BMC Medicine [Internet]. 2018 [cited 2020 Jan 29];16(95). Available from: https://www.ncbi.nlm.nih.gov/pmc/articles/PMC6009054/. 10.1186/s12916-018-1089-4 PMC6009054 29921272

[37] Paul E, Brown GW, Ridde V (2020). COVID-19: time for paradigm shift in the nexus between local, national and global health. BMJ Glob Health.

[38] Norton R (2008). Unintended consequences. The concise Encyclopedia of economics.

[39] Bensadoun E. Coronavirus: Trump warns of ‘suicides by thousands’ if U.S. businesses don’t re-open soon. Global News [Internet]. [cited 2020 Jun 10]; Available from: https://globalnews.ca/news/6725264/trudeau-trump-economy-coronavirus/.

[40] Halperin DT. Coping With COVID-19: Learning from Past Pandemics to Avoid Pitfalls and Panic. Global Health: Science and Practice [Internet]. 2020;8(2). Available from: https://www.ghspjournal.org/content/ghsp/early/2020/06/17/GHSP-D-20-00189.full.pdf. 10.9745/GHSP-D-20-00189 PMC7326514 32554522

[41] DeJong C, Wachter RM. The Risks of Prescribing Hydroxychloroquine for Treatment of COVID-19—First, Do No Harm. JAMA Intern Med [Internet]. 2020 Apr 29 [cited 2020 May 13]; Available from: https://jamanetwork.com/journals/jamainternalmedicine/fullarticle/2765360. 10.1001/jamainternmed.2020.1853 32347894

[42] Harvey F. Coronavirus pandemic “will cause famine of biblical proportions.” The guardian [Internet]. 2020; Available from: https://www.theguardian.com/global-development/2020/apr/21/coronavirus-pandemic-will-cause-famine-of-biblical-proportions? CMP=share_btn_tw.

[43] Kalu B. COVID-19 in Nigeria: a disease of hunger. Lancet Respir Med [Internet]. 2020 Apr 29 [cited 2020 May 4]; Available from: https://www.ncbi.nlm.nih.gov/pmc/articles/PMC7190300/. 10.1016/S2213-2600(20)30220-4 PMC7190300 32359414

[44] WHO. A Coordinated Global Research Roadmap [Internet]. 2020 [cited 2020 May 23]. Available from: https://www.who.int/who-documents-detail/a-coordinated-global-research-roadmap.

[45] CIHR. Operating Grant : COVID-19 May 2020 Rapid research funding opportunity [internet]. Research Net 2020. Available from: https://www.researchnet-recherchenet.ca/rnr16/vwOpprtntyDtls.do?all=1&masterList=true&next=1&prog=3309&resultCount=25&sort=program&type=EXACT&view=currentOpps&language=E.

[46] PWIAS. COVID-19 PWIAS Working Groups [Internet]. 2020. Available from: https://pwias.ubc.ca/program/covid-19-pwias-working-groups.

[47] DFG. Call for Multidisciplinary Research into Epidemics and Pandemics in Response to the Outbreak of SARS-CoV-2 [Internet]. 2020. Available from: https://www.dfg.de/en/research_funding/announcements_proposals/2020/info_wissenschaft_20_20/index.html.

[48] Turcotte-Tremblay A-M, Gali Gali IA, De Allegri M, Ridde V (2017). The unintended consequences of community verifications for performance-based financing in Burkina Faso. Soc Sci Med.

[49] Turcotte-Tremblay A-M, De Allegri M, Gali Gali IA, Ridde V. The unintended consequences of combining equity measures with performance-based financing in Burkina Faso. International Journal for Equity in Health. 2018;17(109). 10.1186/s12939-018-0780-6 PMC6151907 30244685

[50] Turcotte-Tremblay A-M, Gali Gali IA, Ridde V. An exploration of the unintended consequences of performance-based financing in 6 primary healthcare facilities in Burkina Faso. Int J Health Policy Manag. 2020 Jun;23. 10.34172/ijhpm.2020.83 PMC9278611 32610814

[51] Sieleunou I, Turcotte-Tremblay A-M, De Allegri M, Fotso J-CT, Yumo HA, Tamga DM, et al. How does performance-based financing affect the availability of essential medicines in Cameroon? A qualitative study. Health Policy & Planning. 2019;1(34):iii4–19. 10.1093/heapol/czz084 PMC6901074 31816071

[52] Turcotte-Tremblay A-M. The Unintended Consequences of a Complex Intervention Combining Performance-Based Financing with Health Equity Measures in Burkina Faso. [Montreal]: Université de Montréal; 2020.

[53] Patton MQ (2011). Developmental evaluation applying complexity concepts to enhance innovation and use.

[54] Morell JA (2010). Evaluation in the face of uncertainty.

[55] Olivier de Sardan J-P, Tidjani Alou A. Epistemology, Fieldwork, and Anthropology. New York: Palgrave Macmillan; 2015.

[56] Patton MQ (2015). Qualitative Research & Evaluation Methods: integrating theory and practice.

[57] Crozier M, Friedberg MW (1977). L’acteur et le système. Les contraintes de l’action collective.

[58] Morin E, Le Moigne J-L. L’intelligence de la complexité. L’Harmattan Paris:France. 1999.

[59] Tremblay M-C, Richard L, Brousselle A, Beaudet N (2014). Learning reflexively from a health promotion professional development program in Canada. Health Promot Int.

[60] Alexander SA, Jones CM, Tremblay M-C, Beaudet N, Rod MH, Wright MT (2020). Reflexivity in health promotion: a typology for training. Health Promot Pract.

[61] Guba EG. Criteria for assessing the trustworthiness of naturalistic inquiries. ECTJ [Internet]. 1981 [cited 2019 Sep 19];29(75). Available from: https://doi.org/10.1007/BF02766777.

[62] Jabeen S (2018). Unintended outcomes evaluation approach: a plausible way to evaluate unintended outcomes of social development programmes. Evaluation and Program Planning..

[63] Public Health Agency of Sweden. Covid-19 in schoolchildren. A comparison between Finland and Sweden [Internet]. p. 15. Report No.: 20108–1. Available from: https://www.folkhalsomyndigheten.se/publicerat-material/publikationsarkiv/c/covid-19-in-schoolchildren/.

[64] Masonbrink AR, Hurley E. Advocating for Children During the COVID-19 School Closures. Pediatrics [Internet]. 2020 Jun 1 [cited 2020 Jul 29]; Available from: https://pediatrics.aappublications.org/content/early/2020/06/15/peds.2020-1440. 10.1542/peds.2020-1440 32554517

[65] McMahon SA, Musoke DK, Wachinger J, Nakitende A, Amongin J, Nanyiri E, Turcotte-Tremblay A-M, Oldenburg C, Bärnighausen T, Ortblad K. Unintended uses, meanings, and consequences: HIV self-testing among female sex workers in urban Uganda. AIDS Care. 2020. Available from: https://europepmc.org/article/med/33138623. 10.1080/09540121.2020.1837722 PMC8088444 33138623

[66] Lubbock J. The beauties of nature and the wonders of the world we live in: Kirk Press; 2011. 464 p.

[67] Chow A, Hein AA, Kyaw WM. Unintended Consequence: Influenza plunges with public health response to COVID-19 in Singapore. Journal of Infection [Internet]. 2020 Apr 30 [cited 2020 May 11]; Available from: http://www.sciencedirect.com/science/article/pii/S0163445320302620. 10.1016/j.jinf.2020.04.035 PMC7192079 32360879

[68] Schrock CR, Montana MC. Rapid COVID-19–related Clinical Adaptations and Unanticipated Risks. Anesthesiology [Internet]. 2020 Apr 21 [cited 2020 May 11]; Available from: https://www.ncbi.nlm.nih.gov/pmc/articles/PMC7176265/. 10.1097/ALN.0000000000003333 PMC7176265 32282425

[69] Kansagra AP, Goyal MS, Hamilton S, Albers GW. Collateral effect of Covid-19 on stroke evaluation in the United States. N Engl J Med. 2020 May;8. 10.1056/NEJMc2014816 PMC7233187 32383831

[70] Lippi G, Henry BM, Bovo C, Sanchis-Gomar F. Health risks and potential remedies during prolonged lockdowns for coronavirus disease 2019 (COVID-19). Diagnosis (Berl). 2020 26;7(2):85–90. 10.1515/dx-2020-0041 32267243

[71] Pietrobelli A, Pecoraro L, Ferruzzi A, Heo M, Faith M, Zoller T, et al. Effects of COVID-19 Lockdown on Lifestyle Behaviors in Children with Obesity Living in Verona, Italy: A Longitudinal Study. Obesity (Silver Spring). 2020 Apr 30;. 10.1002/oby.22861 PMC7267384 32352652

[72] Brown EE, Kumar S, Rajji TK, Pollock BG, Mulsant BH. Anticipating and Mitigating the Impact of the COVID-19 Pandemic on Alzheimer’s Disease and Related Dementias. The American Journal of Geriatric Psychiatry [Internet]. 2020 Apr 18 [cited 2020 May 13]; Available from: http://www.sciencedirect.com/science/article/pii/S1064748120302943. 10.1016/j.jagp.2020.04.010 PMC7165101 32331845

[73] Iaboni A, Mulsant BH (2020). Why do older adults taking antidepressants fall?. Am J Geriatr Psychiatry.

[74] Pather N, Blyth P, Chapman JA, Dayal MR, Flack NAMS, Fogg QA, et al. Forced disruption of anatomy education in Australia and New Zealand: an acute response to the Covid-19 pandemic. Anat Sci Educ. 2020 Apr;18. 10.1002/ase.1968 PMC7264523 32306555

[75] Shuman AG, Campbell BH. Ethical framework for head and neck cancer care impacted by COVID-19. Head & Neck [Internet]. 2020 [cited 2020 May 12];n/a(n/a). Available from: https://onlinelibrary.wiley.com/doi/abs/10.1002/hed.26193. 10.1002/hed.26193 PMC7264503 32329948

[76] Jakovljevic M, Bjedov S, Jaksic N, Jakovljevic I (2020). COVID-19 Pandemia and public and global mental health from the perspective of Global Health Securit. Psychiatr Danub.

[77] Choi KR, Heilemann MV, Fauer A, Mead M (2020). A second pandemic: mental health Spillover from the novel coronavirus (COVID-19). J Am Psychiatr Nurses Assoc.

[78] Ivanov D (2020). Predicting the impacts of epidemic outbreaks on global supply chains: a simulation-based analysis on the coronavirus outbreak (COVID-19/SARS-CoV-2) case. Transp Res E Logist Transp Rev.

[79] Zhang SX, Huang H, Wei F (2020). Geographical distance to the epicenter of Covid-19 predicts the burnout of the working population: ripple effect or typhoon eye effect?. Psychiatry Res.

[80] Kim AHJ, Sparks JA, Liew JW, Putman MS, Berenbaum F, Duarte-García A, et al. A Rush to Judgment? Rapid Reporting and Dissemination of Results and Its Consequences Regarding the Use of Hydroxychloroquine for COVID-19. Ann Intern Med [Internet]. 2020 Mar 30 [cited 2020 May 11]; Available from: https://www.ncbi.nlm.nih.gov/pmc/articles/PMC7138335/. 10.7326/M20-1223 PMC7138335 32227189

[81] Giudicessi JR, Noseworthy PA, Friedman PA, Ackerman MJ. Urgent Guidance for Navigating and Circumventing the QTc-Prolonging and Torsadogenic Potential of Possible Pharmacotherapies for Coronavirus Disease 19 (COVID-19). Mayo Clinic Proceedings [Internet]. 2020 Apr 7 [cited 2020 May 12]; Available from: http://www.sciencedirect.com/science/article/pii/S002561962030313X. 10.1016/j.mayocp.2020.03.024 PMC7141471 32359771

[82] Dore B. Covid-19: collateral damage of lockdown in India. BMJ [Internet]. 2020 Apr 30 [cited 2020 May 10];369. Available from: https://www.bmj.com/content/369/bmj.m1711. 10.1136/bmj.m1711 32354699

[83] Douglas M, Katikireddi SV, Taulbut M, McKee M, McCartney G. Mitigating the wider health effects of covid-19 pandemic response. BMJ [Internet]. 2020 Apr 27 [cited 2020 May 7];369. Available from: https://www.bmj.com/content/369/bmj.m1557. 10.1136/bmj.m1557 PMC7184317 32341002

[84] de Zwart F (2015). Unintended but not unanticipated consequences. Theor Soc.

[85] Ridde V, Pérez D, Robert E (2020). Using implementation science theories and frameworks in global health. BMJ Glob Health.

[86] Bloomrosen M, Starren J, Lorenzi NM, Ash JS, Patel VL, Shortliffe EH (2011). Anticipating and addressing the unintended consequences of health IT and policy: a report from the AMIA 2009 health policy meeting. J Am Med Inform Assoc.

[87] Okeowo A, Degenhardt L. COVID19 and Police Brutality in Africa. WITNESS Blog [Internet]. 2020 [cited 2020 Jun 17]; Available from: https://blog.witness.org/2020/04/covid19-and-police-brutality-in-africa/.

[88] Brooks SK, Webster RK, Smith LE, Woodland L, Wessely S, Greenberg N (2020). The psychological impact of quarantine and how to reduce it: rapid review of the evidence. Lancet.

[89] Bonwitt J, Dawson M, Kandeh M, Ansumana R, Sahr F, Brown H (2018). Unintended consequences of the “bushmeat ban” in West Africa during the 2013-2016 Ebola virus disease epidemic. Soc Sci Med.

[90] Faherty LJ, Doubeni CA (2015). Unintended consequences of screening for Ebola. Am J Public Health.

[91] Chan CC, Chan KHW, Chow CB (2004). Community response to SARS in Hong Kong. Asia Pacific journal of social work and development volume 14, 2004 - issue 1: SARS and. Soc Work.

[92] Perrin PC, McCabe OL, Everly GS, Links JM (2009). Preparing for an influenza pandemic: mental health considerations. Prehospital and Disaster Medicine.

[93] Lau JTF, Yang X, Tsui HY, Pang E, Wing YK (2006). Positive mental health-related impacts of the SARS epidemic on the general public in Hong Kong and their associations with other negative impacts. J Inf Secur.

[94] Dugal M. COVID-19 : il est normal de perdre la notion du temps, car nous n’avons plus de repères [Internet]. Radio-Canada. 2020 [cited 2020 May 22]. Available from: https://ici.radio-canada.ca/premiere/emissions/moteur-de-recherche/segments/entrevue/165361/temporel-coronavirus-quand-jours-semaine.

[95] Janghorban R, Latifnejad Roudsari R, Taghipour A (2014). Skype interviewing: the new generation of online synchronous interview in qualitative research. Int J Qual Stud Health Well-being.

[96] Krouwel M, Jolly K, Greenfield S. Comparing Skype (video calling) and in-person qualitative interview modes in a study of people with irritable bowel syndrome - an exploratory comparative analysis. BMC Med Res Methodol. 2019 29;19(1):219. 10.1186/s12874-019-0867-9 PMC6883529 31783797

[97] Gibson K. Bridging the digital divide: reflections on using WhatsApp instant messenger interviews in youth research. Qual Res Psychol 2020 Apr 17;0(0):1–21.

[98] Lupton D (2020). Doing fieldork in a pandemic [internet].

[99] TMG-Think Tank for Sustainability. Video diaries from Nairobi: Navigating food insecurity in times of the COVID-19 pandemic [Internet]. Medium. 2020. Available from: https://medium.com/enabling-sustainability/video-diaries-from-nairobi-navigating-food-insecurity-in-times-of-the-covid-19-pandemic-296b300b0867.

[100] Kozinets R (2015). Netnography: redefined. 2 édition.

[101] Pink S, Horst H, Postill J, Hjorth L, Lewis T (2015). Tacchi J.

[102] Miller D (2020). How to conduct an ethnography during social isolation.

[103] Salmons J. Participant Observation: How does it work online? [Internet]. MethodSpace. 2020 [cited 2020 May 20]. Available from: https://www.methodspace.com/participant-observation-how-does-it-work-online/.

[104] Penfold RB, Zhang F (2013). Use of interrupted time series analysis in evaluating health care quality improvements. Acad Pediatr.

[105] Quesnel-Vallé A (2020). Collateral damage: the unintended consequences of COVID-19.

[106] UNFPA. COVID-19: A Gender Lens [Internet]. [cited 2020]. Available from: /resources/covid-19-gender-lens.

[107] Clinton C, Sridhar D. The Unintended Consequences of Covid-19 Put Kids at Risk. Elemental [Internet]. 2020 [cited 2020 Jun 11]; Available from: https://elemental.medium.com/the-unintended-consequences-of-covid-19-put-kids-at-risk-d088f486b5b3.

[108] Yaya S, Yeboah H, Charles CH, Otu A, Labonte R (2020). Ethnic and racial disparities in COVID-19-related deaths: counting the trees, hiding the forest. BMJ Glob Health.

[109] Carillon S. Peur et résilience : paroles d’immigrés confinés en situation de précarité. The Conversation [Internet]. 2020 [cited 2020 Jul 30]; Available from: http://theconversation.com/peur-et-resilience-paroles-dimmigres-confines-en-situation-de-precarite-137926.

[110] Ribeiro C. “Pink-collar recession”: how the Covid-19 crisis could set back a generation of women. the Guardian [Internet]. 2020 [cited 2020 Jun 11]; Available from: http://www.theguardian.com/world/2020/may/24/pink-collar-recession-how-the-covid-19-crisis-is-eroding-womens-economic-power.

[111] Woodward EN, Singh RS, Ndebele-Ngwenya P, Melgar Castillo A, Dickson KS, Kirchner JE. A Practical Guide to Incorporating Health Equity Domains in Implementation Frameworks. Implementation Science [Internet]. 2020 Jun 9 [cited 2020 Jun 11]; Available from: https://www.researchsquare.com/article/rs-32704/v1. 10.1186/s43058-021-00146-5 PMC8178842 34090524

[112] Ridgway L (2020). Collateral damage: the unintended consequences of COVID-19.

[113] Mac-Seing M, Zinszer K, Eryong B, Ajok E, Ferlatte O, Zarowsky C (2020). The intersectional jeopardy of disability, gender and sexual and reproductive health: experiences and recommendations of women and men with disabilities in northern Uganda. Sexual and Reproductive Health Matters..

[114] MacKinnon J, Bremshey A (2020). Perspectives from a webinar: COVID-19 and sexual and reproductive health and rights. Sexual and Reproductive Health Matters.

[115] Turcotte-Tremblay A-M, Mc S-CE (2018). A reflection on the challenge of protecting confidentiality of participants while disseminating research results locally. BMC Medical Ethics.

[116] Klein A. La pandémie en science-fiction [Internet]. Historien.ne.s de la santé. 2020. Available from: http://histoiresante.blogspot.com/2020/05/la-pandemie-en-science-fiction.html.

[117] WHO. Social Stigma associated with COVID-19 [Internet]. 2020. Available from: https://www.unicef.org/documents/social-stigma-associated-coronavirus-disease-covid-19.

[118] United Nations. COVID-19 and Human Rights. We are all in this together [Internet]. 2020. Available from: https://www.un.org/victimsofterrorism/sites/www.un.org.victimsofterrorism/files/un_-_human_rights_and_covid_april_2020.pdf.

[119] UN Women. Issue brief: COVID-19 and ending violence against women and girls | Digital library: Publications [Internet]. 2020 [cited 2020 Jul 3]. Available from: https://www.unwomen.org/-/media/headquarters/attachments/sections/library/publications/2020/issue-brief-covid-19-and-ending-violence-against-women-and-girls-en.pdf?la=en&vs=5006.

[120] Rastegar Kazerooni A, Amini M, Tabari P, Moosavi M. Peer mentoring for medical students during COVID-19 pandemic via a social media platform. Med Educ. 2020 Apr;30. 10.1111/medu.14206 PMC7267157 32353893

[121] Saba M. Confinement: un programme d’exercice pour les aînés. La Presse [Internet]. 2020 [cited 2020 May 25]; Available from: https://www.lapresse.ca/covid-19/202005/12/01-5273276-confinement-un-programme-dexercice-pour-les-aines.php.

[122] Narasimha VL, Shukla L, Mukherjee D, Menon J, Huddar S, Panda UK, et al. Complicated alcohol withdrawal-an unintended consequence of COVID-19 lockdown. Alcohol Alcohol. 2020 May;13. 10.1093/alcalc/agaa042 PMC7239212 32400859

