# Supplementary material for: The unintended consequences of COVID-19 mitigation measures matter: practical guidance for investigating them
Source: BMC Med Res Methodol. 2021 Feb 10;21:28. doi: 10.1186/s12874-020-01200-x (PMC7873511; doi:10.1186/s12874-020-01200-x)
Supplement: Supplementary file 1 — Additional file 1. [file 12874_2020_1200_MOESM1_ESM.docx]

| Additional file 1. Examples of unintended consequences of COVID-19 mitigation measures found in the literature | | |
| --- | --- | --- |
| Sector | **Unintended consequences** | **Brief description** |
| Academia / education | Increase in academic inequalities | - In the UK, 64% of secondary pupils in state schools from the richest households are being offered active help from schools, compared with 47% from the poorest fifth of families (1). **Children from better-off families are spending 30% more time on home learning than are those from poorer families** (1)**.** - Women account for about one-third of all authors who published papers related to COVID-19 since the beginning of the outbreak in January 2020. Women’s representation is lower still for first and last authorship positions (2). The percentage of women as first author was higher in journals with impact factor above 7 in comparison with those with impact factor below 2. |
|  | Concerns over career prospects | - In Iran, medical students experiencing unintended pauses in their education due to university closures find themselves coping with mental and emotional issues, including stress, anxiety, and fear (3). - In Canada, 11% of students were not able to complete credentials as planned, and 48% of students lost their job or were temporarily laid off. Over 40% of students were very or extremely worried about their studies, financial situation, and jobs (4). - In the USA, early career scientists experienced delayed graduation, fewer experiments, delayed publications, and lost job opportunities (5). |
| Agriculture and food | Food insecurity | - More than 30 countries could experience widespread famine (6). |
| Economy | Changes in prices | - Coronavirus lockdowns contributed to plunging oil price (7). |
|  | Reduction in international aid | - Oxfam International withdrew from 18 countries and laid off almost one-third of its program staff amid financial pressures brought on by the pandemic (8). |
| Environ-ment | Changes in environmental outcomes such as air quality, waste, and recycling | - In countries such as China, USA, Italy, and Spain, contingency policies were linked to improved air quality, clean beaches, and less environmental noise (9). However, increased waste and reduction of recycling are negative side effects of COVID-19 (9). - Contingency policies have led to reduced pollution and re-emerging wildlife (7). - In China, air pollutant levels plummeted during the national lockdown in February, bottomed out in early March, and have now overshot their pre-crisis levels. Overshoots suggest a “dirty” recovery in which the more highly polluting sectors are leading (10). |
|  | Reduction in seismic noise | - In Belgium, vibrations caused by human activity have fallen by about one-third following the introduction of coronavirus containment measures (11). |
|  | Changes in animal behaviour | - The global shutdown has reduced the vibrations in Earth’s crust, which may be sufficient to modulate neurobehavioral functions in rodents (12). - In the USA, there has been an increase in rodent activity as rodents search for new sources of food (13). |
| Legal | Infringements of human rights | - In India, the use of tracking technology has blurred the lines between what is voluntary versus mandatory and what is privacy-preserving versus privacy-invading (14). |
| Public health | Changes in lifestyle behaviours | - For obese children in the UK, intakes of chips, red meat, and sugary drinks increased during the lockdown (15). Time spent in sports activities decreased by 2.30 hours/week, while screen time increased by 4.85 hours/day. Sleep time increased by 0.65 hours/day (15). - In Canada, there has been an increase in problematic substance use and opioid-related overdose deaths (16). |
|  | Gender-based violence | - Early reports suggest a dramatic increase in domestic violence and disruption of essential gender-based violence services for women and girls when they need them the most (17). - Globally, violence against women and girls has intensified since the outbreak of COVID-19 (18). For example, in Argentina, emergency calls for domestic violence cases have increased by 25% since the lockdown on March 20^th^ (18). |
|  | Reduced access and utilization of other essential healthcare services and products | - In India, there was a dip in emergency heart attack cases since the lockdown. There are fears that patients who are afraid of getting infected may be dying at home (19). Essential services have been reoriented to treat COVID-19 patients (19). Patients who cannot access essential services due to lack of transportation have died (19). - In the Democratic Republic of Congo, a one-track focus on COVID-19 is negatively impacting the measles response, including transporting vaccines, assembling dedicated teams, and launching vaccination campaigns, contributing to a resurgence of measles (20). - In the USA, a decrease in the use of stroke imaging from the pre-pandemic epoch to the early-pandemic epoch was seen across all age, sex, and stroke severity subgroups (21). - Some 13.5 million children have already missed out on vaccination for polio, measles, human papillomavirus, yellow fever, cholera and meningitis since the suspensions began (22). - Although chloroquine and hydroxychloroquine were not proven to be effective to treat COVID-19 patients, some pharmacies in West Africa reported soaring price inflations, stockouts, and the emergence of informal market supply channels (23,24). |
|  | Changes in morbidity and mortality | - In India, there was an increase in the average number of cases of severe alcohol withdrawal syndrome presenting to hospitals following lockdowns that included the closure of alcohol vends (25). - According to a conservative scenario in LMICs, coverage reductions of 9.8–18.5% and wasting increase of 10% over 6 months would result in 253,500 additional child deaths and 12,200 additional maternal deaths (26). - In Nepal, a sharp increase in maternal mortality was observed following the lockdown due to prohibitions on public movement and all non-essential transport, a limitation on healthcare services as hospitals focus on Covid-19, and healthcare providers’ fears of COVID-19 transmission (27). - More children will be paralyzed in countries where polio is still circulating, and the virus will likely spread to countries that are polio-free (22). - The number of "years of life lost" is higher with the interruption in the economy than with an approach similar to that advocated by Sweden, because the victims of COVID-19 are older (423,000 vs. 397,000) (28). - In India, a model suggests that a three-month interruption in the economy would increase the number of tuberculosis deaths by 19% between 2020 and 2025 (28). - In sub-Saharan Africa, confinement may cause an increase in malaria deaths from 50,000 to 500,000 between 2020 and 2025 (28). - In Spain, there have been significant reductions in total visits to the trauma emergency department, workplace accidents, traffic accidents, and hospital admissions during the COVID-19 pandemic (29). - In Singapore, influenza activity took an unprecedented steep decline following the introduction of social distancing measures (30). - In Greece, there was a significant drop in fatal injuries resulting from road traffic accidents in the 2020 period (31). - In the USA, the potential benefits from school closures need to be weighed against costs of health-care worker absenteeism associated with additional child-care obligations. Estimates suggest that if the infection mortality rate of COVID-19 increases from 2.00% to 2.35% when the health-care workforce declines by 15.0%, school closures could lead to a greater number of deaths than they prevent (32). - Nursing home staff experienced moral distress due to the potential harms associated with isolation of residents, as well as the severe consequences if infection control measures are not effectively implemented (33). In the context of staffing shortages, lack of training, and lack of resources, more restrictive interventions place residents of nursing homes (e.g. isolation of residents with dementia) at higher risk of harm, including the risk of falls and injuries, stroke, deconditioning, skin breakdown, blood clots, and death (33). |
|  | Inequitable health outcomes | - In the USA, minorities, especially Black people, have been infected and killed at a disproportionately higher rate (34). - In India, confinement measures may increase proximity among vulnerable populations (35). Due to lack of transportation, workers, many of whom are migrants, will have to walk long journeys to and from work, exposing themselves to police brutality (35). Vulnerable populations in India will lack access to food (35). - In countries like the United Kingdom and the United States, contingency protocols for the management of organ transplants may prioritize distance over equitable sharing principles during the pandemic (36). Some donations operate under stringent criteria based on local capacity. Rates of donation are higher in areas of low social deprivation. This raises concerns about patients from areas of high social deprivation and of minority ethnic status (36). - In the United States, the use of telepsychiatry in response to COVID-19 raises concerns about inadvertently exacerbating mental health disparities, as certain groups, including the poor, the less-educated, and minorities, may not be as likely to use digital services (37). |

References for Additional file 1.

1. Andrew A, Cattan S, Dias MC, Farquharson C, Kraftman L, Krutikova S, et al. Learning during the lockdown: real-time data on children’s experiences during home learning [Internet]. 2020 [cited 2020 May 20]. Available from: https://www.ifs.org.uk/publications/14848

2. Pinho-Gomes A-C, Peters S, Thompson K, Hockham C, Ripullone K, Woodward M, et al. Where are the women? Gender inequalities in COVID-19 research authorship. BMJ Global Health. 2020 Jun 1;5(7):e002922.

3. Rastegar Kazerooni A, Amini M, Tabari P, Moosavi M. Peer mentoring for medical students during COVID-19 pandemic via a social media platform. Med Educ. 2020 Apr 30;

4. Statistics Canada. How are postsecondary students in Canada impacted by the COVID-19 pandemic? [Internet]. 2020. Available from: https://www150.statcan.gc.ca/n1/pub/11-627-m/11-627-m2020032-eng.htm

5. Yan W. Early-career scientists at critical career junctures brace for impact of COVID-19. Science [Internet]. 2020 [cited 2020 May 19]; Available from: https://www.sciencemag.org/careers/2020/04/early-career-scientists-critical-career-junctures-brace-impact-covid-19

6. Harvey F. Coronavirus pandemic “will cause famine of biblical proportions.” The guardian [Internet]. 2020; Available from: https://www.theguardian.com/global-development/2020/apr/21/coronavirus-pandemic-will-cause-famine-of-biblical-proportions?CMP=share_btn_tw

7. Milman O. Pandemic side-effects offer glimpse of alternative future on Earth Day 2020. The Guardian [Internet]. 2020 Apr 22 [cited 2020 May 19]; Available from: https://www.theguardian.com/environment/2020/apr/22/environment-pandemic-side-effects-earth-day-coronavirus

8. Worley W. Exclusive: Oxfam to lay off 1,450 staff and withdraw from 18 countries. Devex [Internet]. 2020; Available from: https://www.devex.com/news/exclusive-oxfam-to-lay-off-1-450-staff-and-withdraw-from-18-countries-97286#.XsVJllYw7K0.twitter

9. Zambrano-Monserrate MA, Ruano MA, Sanchez-Alcalde L. Indirect effects of COVID-19 on the environment. Science of The Total Environment. 2020 Aug 1;728:138813.

10. CREA. China’s air pollution overshoots pre-crisis levels for the first time [Internet]. 2020 p. 14. Available from: https://energyandcleanair.org/wp/wp-content/uploads/2020/05/China-air-pollution-rebound-final.pdf

11. Gibney E. Coronavirus lockdowns have changed the way Earth moves. Nature. 2020 Mar 31;580(7802):176–7.

12. Manda K. Possible challenges in behavioral phenotyping of rodents following COVID-19 lockdown. Lab Animal. 2020 Jun;49(6):159–159.

13. CDC. Coronavirus Disease 2019 (COVID-19). Robent Control [Internet]. Centers for Disease Control and Prevention. 2020 [cited 2020 Jul 2]. Available from: https://www.cdc.gov/coronavirus/2019-ncov/php/rodents.html

14. O’Neill PH. India is forcing people to use its covid app, unlike any other democracy. MIT Technology Review [Internet]. 2020; Available from: https://www.technologyreview.com/2020/05/07/1001360/india-aarogya-setu-covid-app-mandatory/?utm_medium=tr_social&utm_campaign=site_visitor.unpaid.engagement&utm_source=Twitter#Echobox=1589205626

15. Pietrobelli A, Pecoraro L, Ferruzzi A, Heo M, Faith M, Zoller T, et al. Effects of COVID-19 Lockdown on Lifestyle Behaviors in Children with Obesity Living in Verona, Italy: A Longitudinal Study. Obesity (Silver Spring). 2020 Apr 30;

16. ublic Health Agency of Canada. Statement from the Chief Public Health Officer of Canada on COVID-19 [Internet]. Ottawa, Ontario; 2020. Available from: https://www.canada.ca/en/public-health/news/2020/05/statement-from-the-chief-public-health-officer-of-canada-on-covid-198.html

17. John N, Casey SE, Carino G, McGovern T. Lessons Never Learned: Crisis and gender-based violence. Dev World Bioeth. 2020 Apr 8;

18. UN Women. Issue brief: COVID-19 and ending violence against women and girls | Digital library: Publications [Internet]. 2020 [cited 2020 Jul 3]. Available from: https://www.unwomen.org/-/media/headquarters/attachments/sections/library/publications/2020/issue-brief-covid-19-and-ending-violence-against-women-and-girls-en.pdf?la=en&vs=5006

19. Dore B. Covid-19: collateral damage of lockdown in India. BMJ [Internet]. 2020 Apr 30 [cited 2020 May 10];369. Available from: https://www.bmj.com/content/369/bmj.m1711

20. MSF. COVID-19 Must Not Jeopardise the fight against major killer diseases. In DRC, the measles outbreak is far from over [Internet]. 2020. Available from: https://msf.exposure.co/measles-in-drc

21. Kansagra AP, Goyal MS, Hamilton S, Albers GW. Collateral Effect of Covid-19 on Stroke Evaluation in the United States. N Engl J Med. 2020 May 8;

22. Roberts L. Pandemic brings mass vaccinations to a halt. Science. 2020;368(6487):116–7.

23. McBirney S, Baxi S, Kumar KB, Richmond T. The unintended consequences of a proposed cure for COVID-19. TheHill [Internet]. 2020 Apr 29 [cited 2020 May 1]; Available from: https://thehill.com/opinion/healthcare/495248-the-unintended-consequences-of-a-proposed-cure-for-covid-19

24. CORAF. Chloroquine self-medication for the coronavirus: Warning [Internet]. 2020. Available from: https://www.sonar-global.eu/wp-content/uploads/2020/03/CRCF-Note-on-Chloroquine-for-COVID-210320.pdf

25. Narasimha VL, Shukla L, Mukherjee D, Menon J, Huddar S, Panda UK, et al. Complicated Alcohol Withdrawal-An Unintended Consequence of COVID-19 Lockdown. Alcohol Alcohol. 2020 May 13;

26. Roberton T, Carter ED, Chou VB, Stegmuller A, Jackson BD, Tam Y, et al. Early Estimates of the Indirect Effects of the Coronavirus Pandemic on Maternal and Child Mortality in Low- and Middle-Income Countries [Internet]. Rochester, NY: Social Science Research Network; 2020 Apr [cited 2020 May 19]. Report No.: ID 3576549. Available from: https://papers.ssrn.com/abstract=3576549

27. Poudel A. A 200 percent increase in maternal mortality since the lockdown began. The Kathmandu Post [Internet]. 2020 [cited 2020 May 29]; Available from: https://kathmandupost.com/national/2020/05/27/a-200-percent-increase-in-maternal-mortality-since-the-lockdown-began

28. Perreault M. Le dilemme du tiers-monde. La presse [Internet]. 2020; Available from: https://plus.lapresse.ca/screens/46e03782-0f36-4b51-81eb-8ba6e1de7367__7C___0.html?utm_medium=Messenger&utm_campaign=internal%2Bshare&utm_content=screen&fbclid=IwAR1hdeh42Bk-gmH59E6nQksFcAZXc7YjxoFZxFs0zl-IsWod688lQ42Mkfk

29. Nuñez JH, Sallent A, Lakhani K, Guerra-Farfan E, Vidal N, Ekhtiari S, et al. Impact of the COVID-19 Pandemic on an Emergency Traumatology Service: Experience at a Tertiary Trauma Centre in Spain. Injury. 2020 Jul;51(7):1414–8.

30. Chow A, Hein AA, Kyaw WM. Unintended Consequence: Influenza plunges with public health response to COVID-19 in Singapore. Journal of Infection [Internet]. 2020 Apr 30 [cited 2020 May 11]; Available from: http://www.sciencedirect.com/science/article/pii/S0163445320302620

31. Sakelliadis EI, Katsos KD, Zouzia EI, Spiliopoulou CA, Tsiodras S. Impact of Covid-19 lockdown on characteristics of autopsy cases in Greece. Comparison between 2019 and 2020. Forensic Sci Int. 2020 Jun 12;313:110365.

32. Bayham J, Fenichel EP. Impact of school closures for COVID-19 on the US health-care workforce and net mortality: a modelling study. Lancet Public Health. 2020 May;5(5):e271–8.

33. Iaboni A, Cockburn A, Marcil M, Rodrigues K, Marshall C, Garcia MA, et al. Achieving Safe, Effective, and Compassionate Quarantine or Isolation of Older Adults With Dementia in Nursing Homes. Am J Geriatr Psychiatry. 2020 May 4;

34. Yaya S, Yeboah H, Charles CH, Otu A, Labonte R. Ethnic and racial disparities in COVID-19-related deaths: counting the trees, hiding the forest. BMJ Global Health. 2020 Jun 1;5(6):e002913.

35. Sondarjee M. En Inde, le confinement peut tuer. Le Devoir [Internet]. 2020; Available from: https://www.ledevoir.com/opinion/idees/576139/la-ou-le-confinement-peut-tuer

36. Sharma S, Lawrence C, Giovinazzo F. Transplant programmes during COVID-19: Unintended consequences for health inequality. Am J Transplant. 2020;

37. Kannarkat JT, Smith NN, McLeod-Bryant SA. Mobilization of Telepsychiatry in Response to COVID-19-Moving Toward 21st Century Access to Care. Adm Policy Ment Health. 2020 Apr 24;
